# From coughs to complications: the story of Chlamydia pneumoniae

**DOI:** 10.1099/jmm.0.002006

**Published:** 2025-04-25

**Authors:** Florian Tagini, Mirja Puolakkainen, Gilbert Greub, on behalf of the ESCMID Study Group for Mycoplasma and Chlamydia Infections (ESGMAC)

**Affiliations:** 1 Department of Laboratory Medicine and Pathology, Institute of Microbiology, Lausanne University Hospital, Lausanne, Switzerland; 2 Division of Infectious diseases, Department of Medicine, Lausanne University Hospital, Lausanne, Switzerland; 3 Department of Virology, University of Helsinki and Helsinki University Hospital, Helsinki, Finland

**Keywords:** antibiotic susceptibility testing, cell biology, culture, intracellular trafficking, serology

## Abstract

*Chlamydia pneumoniae* is an obligate intracellular bacterium and a significant cause of respiratory infections. It is associated with upper and lower respiratory tract diseases, including bronchitis and pneumonia. The pathogen employs specific virulence factors, such as the Type III Secretion System (T3SS) and Inc proteins, to invade and subvert host cell machinery during its peculiar developmental life cycle. Chronic infections have been linked to asthma and, more controversially, to atherosclerosis and neurodegenerative diseases. Diagnosis primarily relies on PCR-based molecular assays, while treatment includes macrolides, tetracyclines or fluoroquinolones. Despite its clinical relevance, research on *C. pneumoniae* has declined in recent years, highlighting the need for renewed scientific focus.

## Graphical Abstract

Overview of the clinical and diagnostic aspects of *Chlamydia pneumoniae* infections. The graphical representation of the lungs was generated with ChatGPT. 4o mg, milligram; po, per os; iv, intravenously; d, day; qd, quaque die (once a day) bid, bis in die (twice a day).

## Historical perspective

First isolated in 1965 from the conjunctiva of a Taiwanese child during a trachoma vaccine trial, the organism (strain TW-183) was initially cultured in embryonated chicken eggs [1]. With the introduction of cell culture techniques, TW-183 was observed to produce round, dense intracellular inclusions, resembling *Chlamydia psittaci* more than *Chlamydia trachomatis* [1]. A similar strain, IOL-207, was recovered from the eye of an Iranian child in 1967 [2]. Despite these early discoveries, the bacterium’s pathogenic role remained uncertain until the late 1970s when serological analysis linked TW-183 to a pneumonia outbreak in Finland (1977–78), primarily affecting adolescents and young adults [3]. Further evidence emerged when another strain, AR-39, was isolated from respiratory specimens of patients with pneumonia, bronchitis and pharyngitis in Seattle [4]. This led to the fusion of TW-183 and AR-39 under the designation ‘TWAR’ and, in 1989, the official classification of the organism as *Chlamydia pneumoniae* [5,6].

Advances in molecular phylogenetics in 1999 proposed reclassification of *C. pneumoniae* under the genus *Chlamydophila* based on 16S and 23S rRNA sequencing and DNA–DNA hybridization studies [7]. However, subsequent genomic analyses did not support this distinction, and the international subcommittee on the taxonomy of *Chlamydiae* ultimately reaffirmed *Chlamydia pneumoniae* as the accepted name [8,10]. These historical milestones significantly influenced modern diagnostic and therapeutic approaches, improving serological and molecular assays for *C. pneumoniae* detection. Understanding its role in respiratory infections also guided antibiotic treatment strategies, particularly the use of macrolides and tetracyclines, which remain the recommended regimen today.

## Clinical presentations

In humans, *C. pneumoniae* may cause any kind of respiratory tract infection, ranging from upper respiratory tract infections (with symptoms of rhinitis, sore throat or hoarseness), sinusitis or otitis to bronchitis and community-acquired pneumonia. With lower respiratory tract infections, prolonged cough is classically described, with a median duration of 21 days [11,12]. Wheezing is also frequently reported [12]. Overall, pneumonia tends to be mild, especially in children [11,12]. However, the clinical description includes more severe cases that led to hospitalization, with complications such as pleural effusion, empyema and admissions to intensive care units [11,13, 14]. Primary infection in adults may lead to more severe clinical outcomes than reinfection [13]. Also involved in obstructive respiratory diseases, *C. pneumoniae* infection has been associated with asthma initiation, exacerbations, severity and treatment resistance [15,20].

Asymptomatic infections also seem to occur, as *C. pneumoniae* was documented by PCR and culture in healthy individuals [21,22]. Furthermore, it was shown to colonize a fraction of patients with cystic fibrosis, with an unknown contribution to exacerbations [23]. Finally, as outlined above, *C. pneumoniae* has also been reported as an agent of conjunctivitis.

In animals, it is thought to cause a broad range of clinical manifestations, including respiratory, vascular, conjunctival, genitourinary and systemic diseases [24].

## Morbidity

As a trigger of the immune system, there is an association between *C. pneumoniae* infection and reactive arthritis [25,26], myocarditis [27,29], encephalitis [30,31] and Guillain-Barré syndrome [32]. For these rare complications, the exact mechanism remains unclear but may involve molecular mimicry and chronic inflammation, leading to an immune response that mistakenly targets host tissues.

Associations with other chronic diseases have been postulated over the years, notably linking *C. pneumoniae* with atherosclerosis or Alzheimer’s disease. For atherosclerosis, positive serology was strongly linked to coronary heart disease in 1988 [33]. Following this, *C. pneumoniae* was detected in macrophages and the walls of atherosclerotic vessels through culture, PCR and immunohistochemistry methods [34]. Challenging the causality, a randomized controlled trial in 4,372 patients with stable coronary heart disease assessing the effect of short-course clarithromycin treatment in the interventional group led to no favourable impact on outcome [35]. Furthermore, a meta-analysis including 19,217 patients showed no effect of antibiotic treatment on the occurrence of myocardial infection or all-cause mortality [36].

Regarding Alzheimer’s disease, *C. pneumoniae* has been identified by PCR at higher rates post-mortem in affected patients than in controls. This is to be put in perspective with many other pathogens that have been previously linked with Alzheimer’s disease [37]. More research is needed to investigate further the connection between *C. pneumoniae* and Alzheimer’s disease.

## Microbial characteristics

### Phenotypic features

**Fig. 1.**
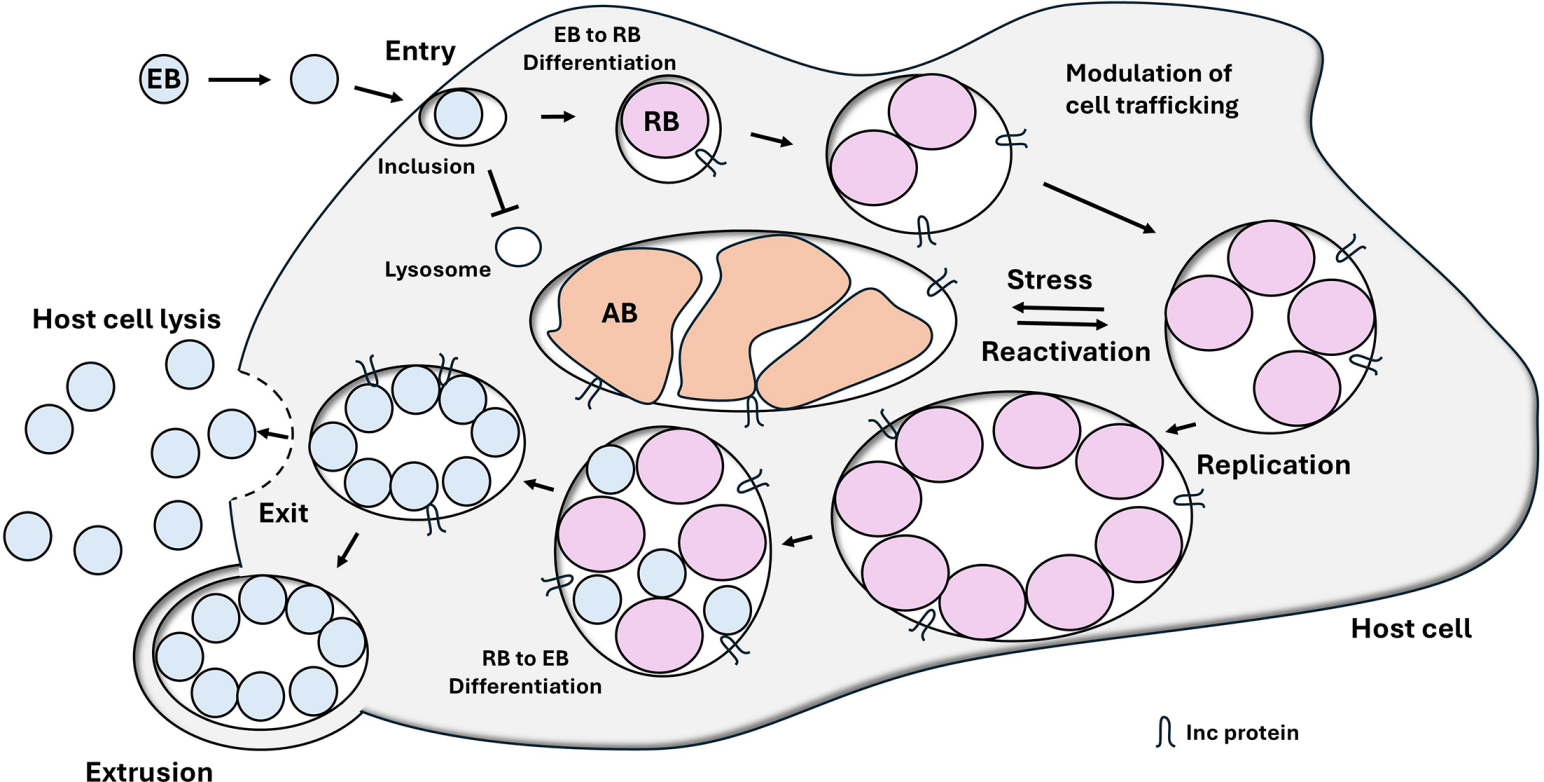
Replicative Cycle of *Chlamydiae* (48–72 h total): EBs are the infectious, extracellular forms that attach to host cells and enter via endocytosis. The compartment containing the EB, called the inclusion, rapidly dissociates from the classic endolysosomal pathway [50]. Pre-synthetized type-III secretion systems effectors are injected into the host cell cytosol to facilitate internalization and prevent apoptosis. Within 2–8 h, EBs differentiate into RBs, the metabolically active, replicative form. Inclusion (Inc) proteins are synthesized and integrated into the inclusion membrane, where they facilitate the redirection of exocytic vesicles from the Golgi apparatus to acquire nutrients and mediate the trafficking of the inclusion towards the microtubule-organizing centre. RBs continue to secrete Type-III secretion system effectors to modulate host cell processes for their own advantage. After replication, RBs transition back into EBs, which are released from the host cell through exocytosis (extrusion) or lysis to initiate a new cycle. Under stressful conditions (e.g. low iron and antibiotic exposure), aberrant bodies (ABs) may form, leading to a persistent, non-replicative state, which may reactivate upon reversal of the stressful condition.

*C. pneumoniae* is an obligate intracellular bacterium. It is characterized by a unique biphasic developmental cycle (Fig. 1), specific to all members of the Chlamydiae (or Chlamydiota) phylum. The cycle comprises two primary forms:

- **Elementary Body** (EB): This is the infectious form of the organism, resistant to environmental stresses. EBs are small (0.2–0.4 µm), metabolically inactive and have a rigid cell wall because of disulphide bonds between a cysteine-rich surface protein, allowing them to survive outside the host cell. EBs adhere to and are engulfed by the cell upon contact with a susceptible host cell, initiating infection.
- **Reticulate Body** (RB): Once inside the host cell, EBs reorganize into RBs, the non-infectious, metabolically active form. RBs are larger and have a more permeable cell membrane, allowing nutrient intake. They replicate by binary fission within a specialized vacuole, termed an inclusion. After multiple replication cycles, RBs condense back into EBs.

Towards the end of its life cycle, newly formed EBs are released from the host cell, either by cell lysis or extrusion, ready to infect new cells. This life cycle is central to *C. pneumoniae'*s ability to cause disease, allowing the bacteria to evade immune detection and establish persistent infections. Under stressful conditions – such as iron deprivation, exposure to interferon-gamma or treatment with certain antibiotics (e.g. beta-lactams) *– C. pneumoniae* can enter a persistent state. In this state, the bacteria transform into aberrant bodies (ABs) [38], which are enlarged, non-replicative forms that allow the pathogen to survive for extended periods without triggering a strong immune response. This persistence is clinically significant because it may contribute to chronic infections and has been implicated in conditions such as persistent respiratory disease.

Structurally, *Chlamydia* species have unique features that differentiate them from other bacteria. *Chlamydia* lipopolysaccharide (LPS) is a truncated form of LPS that lacks the outer core and O-antigen, consisting mainly of lipid A and a unique Kdo (3-deoxy-d-manno-oct-2-ulosonic acid) structure [39]. Unlike other bacteria, *Chlamydia* species contain a distinctive Kdo trisaccharide epitope, αKdo(2→8)-αKdo(2→4)-αKdo, which serves as a specific taxonomic, diagnostic marker and target for vaccine development [39]. From its modified lipid-A structure, chlamydial LPS is less potent in triggering immune responses compared with typical Gram-negative LPS, contributing to immune evasion and persistent infection [40].

Unlike most bacteria, *Chlamydia* has minimal peptidoglycan, a component typically targeted by penicillin. Instead of forming a continuous layer, peptidoglycan accumulates only at mid-cell, where it likely plays a role in bacterial division by interacting with the Tol-Pal complex [41]. The division process in *C. pneumoniae* is also guided by the polymerization of MreB, an actin-like protein recruited to the mid-cell by RodZ [42]. The absence of a peptidoglycan sacculus explains why RBs are so sensitive to osmotic stress and can only divide intracellularly in an osmotically stable environment. The rigid cell wall of elementary bodies that survive outside cells seems to mainly rely on a network of disulphide bonds between cysteine-rich outer membrane proteins unique to the chlamydial species [43].

Metabolically, *Chlamydia* species are obligate intracellular bacteria, meaning they rely entirely on the host cell for energy. They cannot produce their own ATP and instead use an ATP-ADP translocase, a transporter that allows them to import ATP from the host while exporting ADP. This system, which evolved over a billion years ago through gene duplication, enables *Chlamydia* to sustain protein synthesis and replication despite lacking its energy production pathways [44]. Notably, *C. pneumoniae* cannot synthesize or recover tryptophan, making it highly susceptible to the host’s interferon-gamma response, which depletes tryptophan as an immune defence strategy. This metabolic limitation also explains *C. pneumoniae’s* intrinsic resistance to sulphonamides and trimethoprim, which target folic acid synthesis – another pathway it does not possess [24,45].

### Genotypic features

*C. pneumoniae* and other *Chlamydiaceae* have the smallest prokaryotic genome, with a reduced size of about 1.2 Mb [46]. Interacting with the host cells is so important that it makes it an evolutionary constraint to be efficient. Therefore, approximately 10% of *Chlamydiae* genomic sequences encode for type-III secretion system effectors [47]. Type-III secretion system is a syringe-like protein complex that allows *Chlamydiae* to translocate proteins (called effectors) into the host cell [47]. Interestingly, while most *Chlamydiaceae* bear a plasmid contributing to virulence, this is not true for human *C. pneumoniae* strains [48]. Phylogenetic analyses, together with the plasmid loss in humans, indicate an evolutionary recent zoonotic event leading to *C. pneumoniae* infections in humans, while it is believed that the original host could be amphibians (with potentially some intermediate hosts) [48]. Genomic analysis of a strain from Koala and human strains also showed that humans were initially infected zoonotically by an animal isolate, which then adapted to humans, later allowing human-to-human transmission [48].

The *C. pneumoniae* genome, like other *Chlamydia* species, features other significant genetic elements contributing to its virulence, tissue tropism, and survival strategies. The plasticity zone (PZ) of *Chlamydia spp*. varies significantly in size and gene content across species, influencing virulence and host specificity. *C. pneumoniae* is notable for its absence of a cytotoxin gene in the PZ, a trait differentiating it from many other *Chlamydia* species [46].

Moreover, *C. pneumoniae* stands out for its substantial number of inclusion (Inc) proteins, with around 140–147 genes, more than any other *Chlamydiaceae* species [49]. Inc proteins are produced and inserted into the inclusion membrane, where they redirect exocytic vesicles from the Golgi apparatus to supply nutrients and guide the inclusion’s movement towards the microtubule-organizing centre [50]. These Inc proteins are involved in subverting host cellular function and trafficking to enhance bacterial survival within the host.

*C. pneumoniae* possesses a single ribosomal operon [51], which is representative of the entire genus and highlights the genome reduction that has occurred as a result of its intracellular lifestyle. 16S and 23S rRNA gene sequences are widely used for identifying and classifying bacterial species, with standard identity cut-offs of 97%, 95% and 90% to define species, genus and family levels within *Chlamydiales* [7,52]. However, these thresholds often fail to align with established classifications, especially for closely related strains, and applying the same cut-offs to both genes is inappropriate since 23S rRNA is less conserved than 16S. Additionally, rRNA sequence similarity does not always correspond to whole-genome similarity, limiting its accuracy in reflecting true evolutionary relationships [53].

## Laboratory confirmation and safety

### Specimen type

The most common respiratory specimens used for diagnosis are nasopharyngeal swabs or sputa. Nasopharyngeal swabs are acceptable specimen types, and yield tends to be comparable with sputa [54]. However, significant differences in copy numbers per ml may be observed according to the timing of the infection. Lower respiratory tract samples should be preferred when sampled late in the course of the disease. When needed (particularly in case of severe diseases and/or immunosuppression), more invasive respiratory samples such as bronchoalveolar lavage and bronchial aspirates can be obtained and analysed.

### Laboratory confirmation

#### Molecular diagnosis

PCR is a cornerstone of molecular diagnostics, providing a highly accurate and rapid method for pathogen detection. This technique is widely recognized for its excellent analytical sensitivity and high specificity, making it an essential tool in clinical and epidemiological investigations [55,57]. A broad range of commercial and laboratory-developed (home-made) PCR assays are available, offering flexibility in diagnostic applications. These assays can be performed in single-plex format, targeting a single pathogen, or in multiplex format, allowing the simultaneous detection of multiple pathogens within a syndromic framework [55,59]. The gene targets that have been used include species-specific 16S rRNA gene, *ompA* or *pst1* gene [55,56]

Several commercial PCR platforms have been developed for efficient pathogen detection. For example, the BioFire FilmArray Respiratory Panel (bioMérieux) and the Allplex Respiratory Panel (Seegene) are widely used multiplex PCR assays capable of detecting *C. pneumoniae* along with other respiratory pathogens in a single test run. Multiplex PCR assays are particularly valuable for the surveillance and active monitoring of *C. pneumoniae* epidemiology, as they enable the detection of co-circulating pathogens in respiratory infections. Given the frequent occurrence of co-infections with viral or bacterial pathogens [60], careful interpretation of PCR results is crucial to avoid diagnostic pitfalls. The mere presence of microbial DNA does not necessarily indicate active infection, as it may reflect colonization or residual nucleic acids from a previous infection. Therefore, integrating quantitative results can provide additional insights into microbial load, aiding in the differentiation between transient detection and clinically relevant infections. The costs of multiplex PCR assays can be substantial, so their use should be carefully considered in each setting. For example, they may be reserved for patients likely to be hospitalized, where the diagnostic information can significantly impact clinical management.

In clinical practice, combining PCR results with clinical findings, serological data and other laboratory tests remains essential for establishing a definitive diagnosis and guiding appropriate patient management.

#### Serology

While serology has been historically widely used to diagnose *C. pneumoniae* infections, this technique should be discouraged when molecular testing is available. When used, the recommended technique by the Centers for Disease Control and Prevention(CDC) is microimmunofluorescence assays (using whole-cell antigens, typically purified elementary bodies), with criteria of acute infection being IgM titres of ≥1 : 16 or a fourfold increase in IgG [61]. For a definitive diagnosis, a convalescent serum is generally required. Antibodies appear significantly later than the onset of symptoms – 2 to 3 weeks for IgM and 4 to 8 weeks for IgG [61]. The delayed antibody response and the need for convalescent sera render serology impractical for timely diagnosis and treatment.

Furthermore, the assays have variable sensitivity and specificity [62] and, for several reasons, can correlate poorly with the detection of the organism through culture or PCR, especially in children [63]. Several investigators have questioned both the sensitivity and specificity of serology. For example, a CAPNETZ (German Community-Acquired Pneumonia Competence Network) study by Wellinghausen *et al*. found that 17 patients with PCR-confirmed community-acquired pneumonia had negative microimmunofluorescence (MIF) test results [64]. Also, 19% of healthy adults showed serological signs of infection despite negative culture and PCR results [64]. This might be due to the challenging timing of the specimens (taken late when the bacterial load has already decreased) and differences in the performance of the serological assay.

### Culture

For reference centres, isolation in cell culture [61] may be performed to amplify the bacteria for further genomic or antimicrobial susceptibility testing and research purposes. Culture has an elevated turnaround time, requires specialized expertise and suffers from relatively low sensitivity [61]. To isolate the pathogen, specialized cell lines (HL and HEp-2) may be used. Inoculation requires centrifugation onto monolayer of cells. The incorporation of cycloheximide (0.6–0.8 µg ml^−1^) into the culture medium, along with small inoculum and slow expansion, increases the chances of successful culture [65]. Finally, positive cultures must be confirmed by PCR or FITC-conjugated antibodies for detection.

### Laboratory safety

Documented asymptomatic infections have been reported after a laboratory accident leading to the inhalation of infectious particles in unmasked workers [66]. BSL-2 practices, containment equipment and facilities are recommended for personnel handling clinical specimens, cultures or other materials potentially containing strains of *C. pneumoniae*. Additionally, ABSL-2 practices, containment equipment and facilities are advised for procedures involving animals experimentally infected with * C. pneumoniae* strains, ensuring rigorous control and preventing contamination or infection spread [67].

## Treatment and resistance

### Treatment

The intracellular localization of *C. pneumoniae* poses challenges to antimicrobial treatment. Drugs can permeate through the outer layers of mammalian cells, but they also need to gain access to the bacterial cytoplasm. For community-acquired pneumonia caused by *C. pneumoniae*, directed therapy is based on microbiologically active drugs such as macrolides (azithromycin or clarithromycin), tetracycline (doxycycline) or fluoroquinolones (levofloxacin or moxifloxacin). Some experts recommend azithromycin as a first-line treatment because of the shorter duration of administration (5day regimen) and favourable toxicity profile [68]. Ten day regimens of clarithromycin (500 mg twice daily), doxycycline (100 mg twice daily), levofloxacin (500 or 750 mg once daily) or moxifloxacin (400 mg once daily) seem to be acceptable therapeutic options, while there are expert opinions regarding the duration of the antimicrobial therapy, with proposed longer durations of up to 21 days [69]. While treating acute asthma exacerbations caused by *C. pneumoniae* is supported by evidence, there are controversies regarding the need to treat asthmatic patients without signs of acute infection [69]. There are currently no other chronic conditions where *C. pneumoniae* eradication should be considered.

For paediatric patients, macrolides are also usually first-line treatment with erythromycin suspension at a dose of 50 mg kg^–1^ day^–1^ for a duration of 10 to 14 days, clarithromycin suspension at 15 mg kg^–1^ day^–1^ for 10 days, or azithromycin suspension starting with a 10 mg kg^–1^ dose on the first day, then continuing with 5 mg kg^–1^ once daily for the next 4 days. It may be necessary to administer a second course of treatment to some patients [69].

Although no clinical efficacy trial has been performed, several studies showed macrolides or quinolones to successfully eradicate (in 70–86%) *C*. *pneumoniae* from the nasopharynx of patients with community-acquired pneumonia [70,73]. Similar to *Mycoplasma pneumoniae*, the organism may persist in the body even after treatment. The clinical significance of this is not known.

### Resistance

Due to the need for reference centre testing for antimicrobial susceptibility testing, routine testing is not performed, and thus, data on antibiotic susceptibility of *C. pneumoniae* strains remain scarce. Furthermore, most available data have been obtained more than 20 years ago [74]. These data indicated susceptibilities to macrolides, tetracyclines and fluoroquinolones with low minimally inhibitory concentrations. No resistant strain arose from exposure to subinhibitory antibiotic concentrations [75]. There is currently no report of resistant isolates, and treatment failures could result from subinhibitory antibiotic concentrations in the tissue. Although current reports show no significant resistance to *C. pneumoniae*, the widespread use of macrolides and quinolones in clinical settings does raise concerns about the potential for emerging resistance. Over time, the constant exposure of the bacterium to these antibiotics could still select for resistant strains, making future treatment more challenging. In the latest developments, a new molecule called nafithromycin showed good *in vitro* activity on *C. pneumoniae* [76]. Microbiological efficacy in clinical trials remains to be established.

## Pathogenic strategies

### Host range

*C. pneumoniae* has been identified in humans, marsupials (koalas, bandicoots, potoroos), horses, reptiles (snakes, iguanas, chameleons) and amphibians (frogs, turtles) [77].

### Virulence factors

Many virulence factors are conserved across the *Chlamydiaceae* family, enabling these obligate intracellular bacteria to invade, survive and replicate within host cells.

A pivotal virulence factor is its type III secretion system (T3SS), which translocates effector proteins into the host cell, manipulating host cell functions to promote bacterial entry, survival and replication [47]. This system is essential for maintaining a protective intracellular niche and averting lysosomal degradation. One such effector is SemD, which interacts with the host cell’s plasma membrane and recruits phosphatidylinositol 4-kinase IIIβ, producing phosphatidylinositol 4-phosphate. This lipid modification is crucial for creating a favourable environment for bacterial replication [78]. Another effector, CopB, is believed to play a role in forming pores in the host cell membrane, facilitating the translocation of other effector proteins into the host cytosol [79]. Additionally, the T3SS-related protein CPn0809 has been implicated in the translocation process, although its exact function remains to be fully elucidated [80].

Polymorphic membrane proteins (Pmps) are a prominent family of autotransporter proteins in *C. pneumoniae*, comprising up to 21 members – the largest protein family in this species [81]. These surface-exposed proteins are characterized by signature repeating FxxN and GGA(I, V, L) tetrapeptide motifs [82]. Pmps play crucial roles in the pathogenesis of *C. pneumoniae*. They are involved in adhesion to host cells, facilitating bacterial entry and colonization. Additionally, certain Pmps have been implicated in triggering inflammatory responses in human endothelial cells, contributing to the bacterium’s ability to cause disease.

*C. pneumoniae* can also adopt a persistent state under adverse conditions, transforming its morphology (e.g. aberrant bodies) and metabolism to resist antimicrobial treatments and immune responses, potentially contributing to chronic infection states.

These virulence factors are instrumental for *C. pneumoniae’s* disease-causing capabilities, immune system evasion and persistence within the host, contributing to both acute and potentially chronic health complications.

### Host response

*C. pneumoniae* triggers a host immune response by infecting respiratory epithelial cells, where it induces pro-inflammatory cytokine production, such as IL-6 and TNF-α, to recruit immune cells. The pathogen evades host defences by persisting within macrophages, allowing chronic infection and potential tissue damage. Adaptive immunity involves a delayed but crucial Th1 response, with IFN-γ playing a key role in controlling bacterial replication [83].

## Epidemiology

### Transmission (routes and modes)

*C. pneumoniae* is primarily transmitted through respiratory droplets when an infected person coughs or sneezes. Transmission through aerosols is likely to occur, although few studies are available. *C. pneumoniae* was shown to survive small particle aerosolization [84,85] and remained infectious in a mice model [85]. Close contact with infected individuals or contact with contaminated surfaces followed by touching the nose or mouth could also facilitate transmission. For instance, *C. pneumoniae* can survive on some inanimate surfaces for up to 30 h [85].

### Infection (mechanisms, infectious doses/infectivity rate)

The bacterium infects the respiratory tract, attaching to and entering epithelial cells. It then multiplies within the host cells, leading to cell death and inflammation. The infectious dose is not well defined but is believed to be relatively low, given its ability to cause outbreaks in closed populations. The infectivity of *C. pneumoniae* varies with the cell type and experimental conditions in human cell lines, while in animals, 100–1,000 inclusion-forming units are sufficient to cause infections [86].

### Epidemiology (incidence, endemic regions, affected populations)

*C. pneumoniae* infections are widespread globally and are responsible for a significant proportion of community-acquired pneumonia. Infections affect all age groups but are more common in children from school age onward. According to seroepidemiological studies, primary/first infections are unlikely to occur in temperate countries before the age of five [5]. Reinfections tend to occur more frequently in older adults (>65 years old), who are also at higher risk of severe diseases. *C. pneumoniae* infections have generally good outcomes with low mortality (<2%) [11,12]. It is endemic worldwide, and outbreaks can occur in community settings like schools and military bases. Interestingly, during the COVID-19 pandemic, the circulation of *C. pneumoniae* decreased in most of the European centres surveyed (*n*=20) in a recent study. The number of detections dramatically increased in the second half of 2023 and was statistically significant in Switzerland and Slovenia [54, Tagini *et al*., article in press]. Notably, significant geographical variations were observed between different regions. This was speculated to come from a reduced immunity from limited exposure to diverse strains over 2020–2022 due to SARS-CoV-2 containment efforts and relaxed hygiene practices post-pandemic. There is no foreseen impact of global warming on *C. pneumoniae* epidemiology.

### Risk groups (population, disease status, human genetic traits, comorbidities)

While everyone is susceptible, individuals in crowded environments face an increased likelihood of exposure and outbreaks. High-risk settings include college residence halls, correctional and detention facilities, hospitals, long-term care facilities, military training centres, and schools. The proximity of individuals in these environments facilitates the spread of the infection, making preventive measures and early detection crucial in reducing transmission and severe outcomes. Risk factors for more severe disease include age above 65 years old and may also include immunosuppression, as well as chronic lung diseases, although it has never been demonstrated in clinical studies.

### Prevention (include if any vaccines are available and if there are any vaccine candidates)

Preventive measures include good respiratory hygiene, like covering the mouth while coughing and regular hand washing or disinfection. For hospitalized patients, most recommendations suggest that standard precautions may be sufficient. In contrast, droplet precautions should be considered in specific settings, at least when there is evidence of intra-facility transmissions.

Currently, there is no vaccine available for *C. pneumoniae*. Developing a vaccine for *C. pneumoniae* is challenging due to its intracellular lifestyle, immune evasion mechanisms, and ability to establish persistent infections. Natural immunity is often weak, and identifying effective antigens remains challenging, with concerns about potential autoimmune reactions. Limited animal models and a lack of strong commercial incentives further hinder vaccine development.

Testing for *C. pneumoniae* enables the identification and treatment of infected individuals, helping likely to prevent further transmission to others.

## Open questions

- What is the current state of antimicrobial susceptibility, including phenotypic and genotypic characteristics?
- How do the genomic variations and dynamics of *C. pneumoniae* evolve during outbreaks?
- Which genomic factors contribute to an increase in pathogenicity, and are there specific clades of *C. pneumoniae* associated with higher virulence?
- Can *C. pneumoniae* cause persistent infections, and how are these associated with chronic diseases?
- How does *C. pneumoniae* interact with other co-pathogens during co-infections, and what implications does this interplay have?
- What constitutes the most effective treatment regimen for infections caused by *C. pneumoniae*?
- What are the most effective strategies for preventing the spread of *C. pneumoniae* in community settings?

## Abbreviations

ABSL-2: animal biosafety level 2
AD: Alzheimer disease
ATP: adenosine triphosphate
BSL-2: biosafety level 2
EB: elementary body
LPS: lipopolysaccharide
PZ: plasticity zone
RB: reticulate body

## References

[1] Kuo CC, Chen HH, Wang SP, Grayston JT (1986). Identification of a new group of *Chlamydia* psittaci strains called TWAR. J Clin Microbiol.

[2] Dwyer RS, Treharne JD, Jones BR, Herring J (1972). Chlamydial infection. Results of micro-immunofluorescence tests for the detection of type-specific antibody in certain chlamydial infections. Br J Vener Dis.

[3] Saikku P, Wang SP, Kleemola M, Brander E, Rusanen E (1985). An epidemic of mild pneumonia due to an unusual strain of *Chlamydia psittaci*. J Infect Dis.

[4] Grayston JT, Kuo CC, Wang SP, Altman J (1986). A new *Chlamydia psittaci* strain, TWAR, isolated in acute respiratory tract infections. N Engl J Med.

[5] Grayston JT, Campbell LA, Kuo CC, Mordhorst CH, Saikku P (1990). A new respiratory tract pathogen: *Chlamydia pneumoniae* strain TWAR. J Infect Dis.

[6] Grayston JT, Kuo C-C, Campbell LA, Wang S-P (1989). *Chlamydia pneumoniae* sp. nov. for *Chlamydia* sp. strain TWAR. Int J Syst Bacteriol.

[7] Everett KD, Bush RM, Andersen AA (1999). Emended description of the order *Chlamydiales*, proposal of *Parachlamydiaceae* fam. nov. and *Simkaniaceae* fam. nov., each containing one monotypic genus, revised taxonomy of the family *Chlamydiaceae*, including a new genus and five new species, and standards for the identification of organisms. Int J Syst Bacteriol.

[8] Bavoil P, Kaltenboeck B, Greub G (2013). In *Chlamydia* veritas. Pathog Dis.

[9] Pannekoek Y, Qi-Long Q, Zhang Y-Z, van der Ende A (2016). Genus delineation of *Chlamydiales* by analysis of the percentage of conserved proteins justifies the reunifying of the genera *Chlamydia* and *Chlamydophila* into one single genus *Chlamydia*. Pathog Dis.

[10] Greub G (2010). International committee on systematics of prokaryotes subcommittee on the taxonomy of the chlamydiae. Int J Syst Evol Microbiol.

[11] Kauppinen MT, Saikku P, Kujala P, Herva E, Syrjälä H (1996). Clinical picture of community-acquired *Chlamydia pneumoniae* pneumonia requiring hospital treatment: a comparison between chlamydial and pneumococcal pneumonia. Thorax.

[12] Conklin L, Adjemian J, Loo J, Mandal S, Davis C (2013). Investigation of a *Chlamydia pneumoniae* outbreak in a federal correctional facility in Texas. Clin Infect Dis.

[13] Ekman MR, Grayston JT, Visakorpi R, Kleemola M, Kuo CC (1993). An epidemic of infections due to *Chlamydia pneumoniae* in military conscripts. Clin Infect Dis.

[14] Augenbraun MH, Roblin PM, Mandel LJ, Hammerschlag MR, Schachter J (1991). *Chlamydia pneumoniae* pneumonia with pleural effusion: diagnosis by culture. Am J Med.

[15] Hertzen LV, Vasankari T, Liippo K, Wahlström E, Puolakkainen M (2002). *Chlamydia pneumoniae* and severity of asthma. Scand J Infect Dis.

[16] von Hertzen LC (2002). Role of persistent infection in the control and severity of asthma: focus on *Chlamydia pneumoniae*. Eur Respir J.

[17] Hahn DL, Peeling RW, Dillon E, McDonald R, Saikku P (2000). Serologic markers for *Chlamydia pneumoniae* in asthma. Ann Allergy Asthma Immunol.

[18] Hahn DL, McDonald R (1998). Can acute *Chlamydia pneumoniae* respiratory tract infection initiate chronic asthma?. Ann Allergy Asthma Immunol.

[19] Webley WC, Hahn DL (2017). Infection-mediated asthma: etiology, mechanisms and treatment options, with focus on *Chlamydia pneumoniae* and macrolides. Respir Res.

[20] Cho YS, Kim T-B, Lee T-H, Moon K-A, Lee J (2005). *Chlamydia pneumoniae* infection enhances cellular proliferation and reduces steroid responsiveness of human peripheral blood mononuclear cells via a tumor necrosis factor-alpha-dependent pathway. Clin Exp Allergy.

[21] Hyman CL, Roblin PM, Gaydos CA, Quinn TC, Schachter J (1995). Prevalence of asymptomatic nasopharyngeal carriage of *Chlamydia pneumoniae* in subjectively healthy adults: assessment by polymerase chain reaction-enzyme immunoassay and culture. Clin Infect Dis.

[22] Miyashita N, Niki Y, Nakajima M, Fukano H, Matsushima T (2001). Prevalence of asymptomatic infection with *Chlamydia pneumoniae* in subjectively healthy adults. Chest.

[23] Pittet LF, Bertelli C, Scherz V, Rochat I, Mardegan C (2021). *Chlamydia pneumoniae* and *Mycoplasma pneumoniae* in children with cystic fibrosis: impact on bacterial respiratory microbiota diversity. Pathog Dis.

[24] Roulis E, Polkinghorne A, Timms P (2013). *Chlamydia pneumoniae*: modern insights into an ancient pathogen. Trends Microbiol.

[25] Carter JD, Hudson AP (2010). The evolving story of *Chlamydia*-induced reactive arthritis. Curr Opin Rheumatol.

[26] Melby KK, Kvien TK, Glennås A, Anestad G (1999). *Chlamydia pneumoniae* as a trigger of reactive arthritis. Scand J Infect Dis.

[27] Durel C-A, Saison J, Chidiac C, Ferry T (2015). A case of interstitial pneumonia, myocarditis and severe sepsis caused by *Chlamydia pneumoniae*. BMJ Case Rep.

[28] Hoefer D, Poelzl G, Kilo J, Hoermann C, Mueller JL (2005). Early detection and successful therapy of fulminant *Chlamydia pneumoniae* myocarditis. ASAIO J.

[29] Suesaowalak M, Cheung MM, Tucker D, Chang AC, Chu J (2009). *Chlamydophila pneumoniae* myopericarditis in a child. Pediatr Cardiol.

[30] Glaser CA, Honarmand S, Anderson LJ, Schnurr DP, Forghani B (2006). Beyond viruses: clinical profiles and etiologies associated with encephalitis. Clin Infect Dis.

[31] Elargoubi A, Verhoeven PO, Grattard F, Stephan J-L, Richard O (2013). Acute encephalitis associated to a respiratory infection due to *Chlamydophila pneumoniae*. Med Mal Infect.

[32] Haidl S, Ivarsson S, Bjerre I, Persson K (1992). Guillain-barré syndrome after *Chlamydia pneumoniae* infection. N Engl J Med.

[33] Saikku P, Leinonen M, Mattila K, Ekman MR, Nieminen MS (1988). Serological evidence of an association of a novel *Chlamydia*, TWAR, with chronic coronary heart disease and acute myocardial infarction. Lancet.

[34] Campbell LA, Kuo CC, Grayston JT (1998). *Chlamydia pneumoniae* and cardiovascular disease. Emerg Infect Dis.

[35] Hilden J, Lind I, Kolmos HJ, Als-Nielsen B, Damgaard M (2010). *Chlamydia pneumoniae* IgG and IgA antibody titers and prognosis in patients with coronary heart disease: results from the CLARICOR trial. Diagn Microbiol Infect Dis.

[36] Andraws R, Berger JS, Brown DL (2005). Effects of antibiotic therapy on outcomes of patients with coronary artery disease: a meta-analysis of randomized controlled trials. JAMA.

[37] Piekut T, Hurła M, Banaszek N, Szejn P, Dorszewska J (2022). Infectious agents and Alzheimer’s disease. J Integr Neurosci.

[38] Panzetta ME, Valdivia RH, Saka HA (2018). *Chlamydia* persistence: a survival strategy to evade antimicrobial effects *in-vitro* and *in-vivo*. Front Microbiol.

[39] Nguyen HP, Seto NOL, MacKenzie CR, Brade L, Kosma P (2003). Germline antibody recognition of distinct carbohydrate epitopes. Nat Struct Mol Biol.

[40] Wang X, Rockey DD, Dolan BP (2020). *Chlamydia lipooligosaccharide* has varied direct and indirect roles in evading both innate and adaptive host immune responses. Infect Immun.

[41] Jacquier N, Viollier PH, Greub G (2015). The role of peptidoglycan in chlamydial cell division: towards resolving the chlamydial anomaly. FEMS Microbiol Rev.

[42] Jacquier N, Frandi A, Pillonel T, Viollier PH, Greub G (2014). Cell wall precursors are required to organize the chlamydial division septum. Nat Commun.

[43] Christensen S, McMahon RM, Martin JL, Huston WM (2019). Life inside and out: making and breaking protein disulfide bonds in *Chlamydia*. Crit Rev Microbiol.

[44] Greub G, Raoult D (2003). History of the ADP/ATP-translocase-encoding gene, a parasitism gene transferred from a *Chlamydiales* ancestor to plants 1 billion years ago. Appl Environ Microbiol.

[45] Chacko A, Barker CJ, Beagley KW, Hodson MP, Plan MR (2014). Increased sensitivity to tryptophan bioavailability is a positive adaptation by the human strains of *Chlamydia pneumoniae*. Mol Microbiol.

[46] Luu LDW, Kasimov V, Phillips S, Myers GSA, Jelocnik M (2023). Genome organization and genomics in *Chlamydia*: whole genome sequencing increases understanding of chlamydial virulence, evolution, and phylogeny. Front Cell Infect Microbiol.

[47] Betts-Hampikian HJ, Fields KA (2010). The chlamydial type III secretion mechanism: revealing cracks in a tough nut. Front Microbiol.

[48] Myers GSA, Mathews SA, Eppinger M, Mitchell C, O’Brien KK (2009). Evidence that human *Chlamydia pneumoniae* was zoonotically acquired. J Bacteriol.

[49] Lutter EI, Martens C, Hackstadt T (2012). Evolution and conservation of predicted inclusion membrane proteins in *Chlamydiae*. Comp Funct Genomics.

[50] Elwell C, Mirrashidi K, Engel J (2016). *Chlamydia* cell biology and pathogenesis. Nat Rev Microbiol.

[51] Kalman S, Mitchell W, Marathe R, Lammel C, Fan J (1999). Comparative genomes of *Chlamydia pneumoniae* and *C. trachomatis*. Nat Genet.

[52] Domman D, Collingro A, Lagkouvardos I, Gehre L, Weinmaier T (2014). Massive expansion of Ubiquitination-related gene families within the *Chlamydiae*. Mol Biol Evol.

[53] Pillonel T, Bertelli C, Salamin N, Greub G (2015). Taxogenomics of the order *Chlamydiales*. Int J Syst Evol Microbiol.

[54] Tagini F, Opota O, Greub G (2024). *Chlamydia pneumoniae* upsurge at a tertiary hospital, Lausanne, Switzerland. Emerg Infect Dis.

[55] Reischl U, Lehn N, Simnacher U, Marre R, Essig A (2003). Rapid and standardized detection of *Chlamydia pneumoniae* using LightCycler real-time fluorescence PCR. Eur J Clin Microbiol Infect Dis.

[56] Tondella MLC, Talkington DF, Holloway BP, Dowell SF, Cowley K (2002). Development and evaluation of real-time PCR-based fluorescence assays for detection of *Chlamydia pneumoniae*. J Clin Microbiol.

[57] Opota O, Brouillet R, Greub G, Jaton K (2017). Methods for real-time PCR-based diagnosis of *Chlamydia pneumoniae*, *Chlamydia psittaci*, and *Chlamydia abortus* infections in an opened molecular diagnostic platform. Methods Mol Biol.

[58] Murphy CN, Fowler R, Balada-Llasat JM, Carroll A, Stone H (2020). Multicenter evaluation of the biofire filmarray pneumonia/pneumonia plus panel for detection and quantification of agents of lower respiratory tract infection. J Clin Microbiol.

[59] Leber AL, Lisby JG, Hansen G, Relich RF, Schneider UV (2020). Multicenter evaluation of the QIAstat-Dx respiratory panel for detection of viruses and bacteria in nasopharyngeal swab specimens. J Clin Microbiol.

[60] Verkooyen RP, Willemse D, Hiep-van Casteren SC, Joulandan SA, Snijder RJ (1998). Evaluation of PCR, culture, and serology for diagnosis of *Chlamydia pneumoniae* respiratory infections. J Clin Microbiol.

[61] Dowell SF, Peeling RW, Boman J, Carlone GM, Fields BS (2001). Standardizing *Chlamydia pneumoniae* assays: recommendations from the Centers for Disease Control and Prevention (USA) and the Laboratory Centre for Disease Control (Canada). Clin Infect Dis.

[62] Tuuminen T, Palomäki P, Paavonen J (2000). The use of serologic tests for the diagnosis of chlamydial infections. J Microbiol Methods.

[63] Hammerschlag MR (2000). *Chlamydia pneumoniae* and the lung. Eur Respir J.

[64] Wellinghausen N, Straube E, Freidank H, von Baum H, Marre R (2006). Low prevalence of *Chlamydia pneumoniae* in adults with community-acquired pneumonia. Int J Med Microbiol.

[65] Campbell LA, Kuo C-C (2009). Cultivation and laboratory maintenance of *Chlamydia pneumoniae*. Curr Protoc Microbiol.

[66] Hyman CL, Augenbraun MH, Roblin PM, Schachter J, Hammerschlag MR (1991). Asymptomatic respiratory tract infection with *Chlamydia pneumoniae* TWAR. J Clin Microbiol.

[67] Biosafety in Microbiological and Biomedical Laboratories (BMBL) 6th Edition (2023). CDC Laboratory Portal. CDC. https://www.cdc.gov/labs/BMBL.html.

[68] Sharma L, Losier A, Tolbert T, Dela Cruz CS, Marion CR (2017). Atypical pneumonia: updates on *Legionella*, *Chlamydophila*, and *Mycoplasma pneumonia*. Clin Chest Med.

[69] Kohlhoff SA, Hammerschlag MR (2015). Treatment of chlamydial infections: 2014 update. Expert Opin Pharmacother.

[70] Roblin PM, Hammerschlag MR (1998). Microbiologic efficacy of azithromycin and susceptibilities to azithromycin of isolates of *Chlamydia pneumoniae* from adults and children with community acquired pneumonia. Antimicrob Agents Chemother.

[71] Hammerschlag MR, Roblin PM (2000). Microbiological efficacy of levofloxacin for treatment of community-acquired pneumonia due to *Chlamydia pneumoniae*. Antimicrob Agents Chemother.

[72] Hammerschlag MR, Roblin PM (2000). Microbiologic efficacy of moxifloxacin for the treatment of community-acquired pneumonia due to *Chlamydia pneumoniae*. Int J Antimicrob Agents.

[73] Roblin PM, Montalban G, Hammerschlag MR (1994). Susceptibilities to clarithromycin and erythromycin of isolates of *Chlamydia pneumoniae* from children with pneumonia. Antimicrob Agents Chemother.

[74] Hammerschlag MR (1994). Antimicrobial susceptibility and therapy of infections caused by *Chlamydia pneumoniae*. Antimicrob Agents Chemother.

[75] Stamm WE (2000). Potential for antimicrobial resistance in *Chlamydia pneumoniae*. J Infect Dis.

[76] Kohlhoff S, Hammerschlag MR (2021). *In vitro* activity of Nafithromycin (WCK 4873) against *Chlamydia pneumoniae*. Antimicrob Agents Chemother.

[77] Bodetti TJ, Jacobson E, Wan C, Hafner L, Pospischil A (2002). Molecular evidence to support the expansion of the hostrange of *Chlamydophila pneumoniae* to include reptiles as well as humans, horses, koalas and amphibians. Syst Appl Microbiol.

[78] Kocher F, Applegate V, Reiners J, Port A, Spona D (2024). The *Chlamydia pneumoniae* effector SemD exploits its host’s endocytic machinery by structural and functional mimicry. Nat Commun.

[79] Bulir DC, Waltho DA, Stone CB, Liang S, Chiang CKW (2015). *Chlamydia* Outer Protein (Cop) B from *Chlamydia pneumoniae* possesses characteristic features of a type III secretion (T3S) translocator protein. BMC Microbiol.

[80] Engel AC, Herbst F, Kerres A, Galle JN, Hegemann JH (2016). The type III secretion system-related CPn0809 from *Chlamydia pneumoniae*. PLoS One.

[81] Mölleken K, Schmidt E, Hegemann JH (2010). Members of the Pmp protein family of *Chlamydia pneumoniae* mediate adhesion to human cells via short repetitive peptide motifs. Mol Microbiol.

[82] Debrine AM, Karplus PA, Rockey DD (2023). A structural foundation for studying chlamydial polymorphic membrane proteins. Microbiol Spectr.

[83] Porritt RA, Crother TR (2016). *Chlamydia pneumoniae* infection and inflammatory diseases. For Immunopathol Dis Therap.

[84] Theunissen HJ, Lemmens-den Toom NA, Burggraaf A, Stolz E, Michel MF (1993). Influence of temperature and relative humidity on the survival of *Chlamydia pneumoniae* in aerosols. Appl Environ Microbiol.

[85] Falsey AR, Walsh EE (1993). Transmission of *Chlamydia pneumoniae*. J Infect Dis.

[86] de Kruif MD, van Gorp ECM, Keller TT, Ossewaarde JM, ten Cate H (2005). *Chlamydia pneumoniae* infections in mouse models: relevance for atherosclerosis research. Cardiovasc Res.

